# P120: Monitoring hand hygiene compliance and the distribution of MRSA in paediatric wards

**DOI:** 10.1186/2047-2994-2-S1-P120

**Published:** 2013-06-20

**Authors:** D Lary, K Hardie, J Randle, A Clavert

**Affiliations:** 1 School of Molecular Medical Sciences, University of Nottingham, Nottingham, UK; 2 School of Nursing, Midwifery and Physiotherapy, University of Nottingham, Nottingham, UK

## Introduction

Hand hygiene considered being the single most effective measure against the spread of healthcare associated infection, but studies have reported poor hand hygiene compliance among healthcare worker during interaction with patients, contributing to the spread of disease. Numerous interventions have aimed to improve the hand hygiene practices of healthcare workers in healthcare settings, however little attention has been paid to patients’ and their visitors’ hand hygiene.

## Objectives

Measure hand hygiene opportunities taken by HCWs’, patients and visitors to gain a picture of theirs hand hygiene compliance and to examine whether hands of HCWs, patients, visitors and surfaces ‘near touch sites’ act as a reservoir for MRSA.

## Methods

Observation of HCWs’, patients’ and visitors’ hand hygiene compliance was measured over period of 10 weeks across 6 paediatric wards in a teaching hospital. Additionally, swabs were taken from subjects’ hands and surfaces and samples were idntified using molecular identification techniques. Antibiotic susceptibility profiling was applied on *S. aureus* isolates to detect the presence of MRSA. Genetic profiles were evaluated using the *spa*sequence-based typingmethodfor discriminating between isolates to evaluate the average linkage within samples.

## Results

A total of 1891 hand hygiene opportunities observed consisting of 1366 for HCW;525 for patients and visitors. Among HCWs’, doctors showing the highest level of complaince compared to other professions(P<0.001). There was no difference in compliance between patients and visitors (P=0.53). A total of 105 samples were obtained from hand and 92 from surfaces. MRSA was observed in 5% of hands and environmental samples. Moreover, samples collected on the same day, from different hands and surfaces had similar microbial fingerprints and patterns of antibiotic sensitivity.

## Conclusion

Levels of HCW’s hand hygiene compliance found in this study were better than the previously reported. On the other hand, we were unable to draw conclusions about patients’ hand hygiene compliance due to the nature of the clinical environment; however, visitors' compliance was considered to be higher than previous reported studies. Furthermorer, hand and surfaces may act as reservoir for MRSA increasing the risk of HCAI.

## Disclosure of interest

None declared

